# Before the light fades, who blows the whistle? : a narrative review on sports dementia

**DOI:** 10.1192/bjo.2021.772

**Published:** 2021-06-18

**Authors:** Olusegun Sodiya, Ovwigho Edafegwotu, Jide Jeje

**Affiliations:** 1 Tees, Esk and Wear Valleys NHS Foundation Trust; 2 Neuropsychiatric Hospital, Aro; 3 Southern Health NHS Foundation Trust

## Abstract

**Aims:**

Traumatic brain injury is a leading risk factor for degenerative conditions. Although in the past this was believed to affect mostly boxers, recent studies have expanded the at-risk population to include American football players, rugby players, hockey players and other athletes involved in contact sports. Hence, there has been growing interest in the media and the public at large on the short and long term impacts of head trauma in sportspersons. The aim of this study is provide an overview of the impact of traumatic brain injury in contact sports and the link to early onset dementia.

**Method:**

For the purpose of this study we conducted a literature search using PubMed electronic base and Google scholar. The search was made in February 2021 and using the following keywords ‘early onset dementia’, ‘presenile dementia’, ‘traumatic brain injury’, ‘contact sports’, ‘sportsmen’, and ‘athletes’. The search words were used individually and in combination to gather relevant articles. Types of studies included were case reports, case series, cohorts, cross-sectional, editorial and newspaper articles.

**Result:**

Most of the published studies have shown significant associations between repeated head trauma and brain morphological changes evidenced by the presence of myelinated axons, astrocytosis, perivascular neuroinflammation and formation of phosphorylated Tau proteinopathy. These contribute significantly to alterations in axonal functioning and synaptic transmissions which sets the stage for neuronal degeneration. These changes affect both the macroscopic and microscopic structures with consequent neurochemical disturbances and functional deficits which, manifest primarily as executive dysfunction.

**Conclusion:**

Current evidence supports an association between participation in contact sports and neurodegenerative disease, despite the protective aspects of sporting activities. Overall the studies reviewed have shown that brain injury remains a potent risk factor for the early onset dementia seen in sportspersons. Consequently, it is prudent for more proactive and precautionary measures to be put in place to reduce impacts of head injury and to better identify and manage brain injury in sports.

